# Impact of a comprehensive cardiac rehabilitation programme versus coronary revascularisation in patients with stable angina pectoris: study protocol for the PRO-FIT randomised controlled trial

**DOI:** 10.1186/s12872-023-03266-z

**Published:** 2023-05-05

**Authors:** Joyce M. Heutinck, Iris A. De Koning, Tom Vromen, Robert-Jan M. Van Geuns, Dick H.J. Thijssen, Hareld M.C. Kemps, On behalf of the PRO-FIT Research Group, Eddy M. Adang, Johanna M. Geleijnse, Pieter van Gorp, Arnoud W. J. van ‘t Hof, Veronica R. Janssen, Harald T. Jorstad, Roderik A. Kraaijenhagen, Jeroen Lammers, Frits H. A. F. de Man, Gijs J. Nollen, Clara E. E. van Ofwegen-Hanekamp, Steven Onkelinx, Laurence M. L. C. Oostveen, Kit C. B. Roes, Madoka Sunamara, Pim A. L. Tonino

**Affiliations:** 1 Department of Industrial Design, Eindhoven University of Technology, Eindhoven, the Netherlands; 2 Department of Medical BioSciences, Radboud University Medical Centre, Nijmegen, the Netherlands; 3 Department of Cardiology, Maxima Medical Centre, Eindhoven, the Netherlands; 4 Department of Cardiology, Radboud University Medical Centre, Nijmegen, the Netherlands; 5 Research Institute for Sport and Exercise Sciences, Liverpool John Moores University, Liverpool, UK

**Keywords:** Coronary artery disease, Cardiac rehabilitation, Coronary revascularisation, Stable angina pectoris, Chronic coronary syndrome, Percutaneous coronary intervention, Cost-effectiveness

## Abstract

**Background:**

Currently, in the majority of patients with stable angina pectoris (SAP) treatment consists of optimal medical treatment, potentially followed by coronary angiography and subsequent coronary revascularisation if necessary”. Recent work questioned the effectiveness of these invasive procedures in reducing re-events and improving prognosis. The potential of exercise-based cardiac rehabilitation on clinical outcomes in patients with coronary artery disease is well-known. However, in the modern era, no studies compared the effects of cardiac rehabilitation *versus* coronary revascularisation in patients with SAP.

**Methods:**

In this multicentre randomised controlled trial, 216 patients with stable angina pectoris and residual anginal complaints under optimal medical treatment will be randomised to: 1) usual care (i.e., coronary revascularisation), or 2) a 12-month cardiac rehabilitation (CR) programme. CR consists of a multidisciplinary intervention, including education, exercise training, lifestyle coaching and a dietary intervention with a stepped decline in supervision. The primary outcome will be anginal complaints (Seattle Angina Questionnaire-7) following the 12-month intervention. Secondary outcomes include cost-effectiveness, ischemic threshold during exercise, cardiovascular events, exercise capacity, quality of life and psychosocial wellbeing.

**Discussion:**

In this study, we will examine the hypothesis that multidisciplinary CR is at least equally effective in reducing anginal complaints as the contemporary invasive approach at 12-months follow-up for patients with SAP. If proven successful, this study will have significant impact on the treatment of patients with SAP as multidisciplinary CR is a less invasive and potentially less costly and better sustainable treatment than coronary revascularisations.

**Trial registration:**

Netherlands Trial Register, NL9537. Registered 14 June 2021.

**Supplementary Information:**

The online version contains supplementary material available at 10.1186/s12872-023-03266-z.

## Background

Stable angina pectoris (SAP) is a highly common condition in Western society, which is typically caused by atherosclerosis, ultimately causing (partial) occlusion of coronary arteries. To reduce the risk of cardiovascular events and improve quality of life, current treatment of patients with SAP starts with optimisation of medication according to prevailing guidelines [1, 2]. When symptoms persist, patients are often referred for coronary angiography and (if needed) coronary revascularisation. Currently, approximately 48% of all (~ 41.000) percutaneous coronary interventions in the Netherlands is performed in stable coronary artery disease patients [3–5]. Importantly, the effectivity of coronary revascularisation in patients with SAP has been questioned increasingly. In 2014, a meta-analysis of trials investigating the effect of percutaneous coronary intervention (PCI) in SAP patients failed to show a reduction in mortality, myocardial infarctions or revascularisation procedures compared to optimal medical treatment (OMT) [6]. Similarly, a randomised controlled trial comparing PCI with a sham procedure in SAP patients showed no difference in symptoms and exercise capacity [7]. Finally, the recent ISCHEMIA trial in 5179 SAP patients found no difference in 3.2-year mortality and cardiac event rates in SAP patients comparing optimal medical treatment with a routine invasive strategy (PCI or coronary-artery bypass grafting). Although revascularisation did not improve prognosis, a sub-analysis of the ISCHEMIA trial did show a superior improvement in anginal complaints following revascularisation compared to OMT. Hence, reduction of symptoms is the prevailing incentive to continue to perform revascularisations in patients with SAP.

Multidisciplinary cardiac rehabilitation (CR) might be a successful alternative treatment in patients with SAP. CR improves achieving lifestyle targets and medication adherence [3, 8] and may also lead to symptom reduction through its systemic effects on cardiovascular function and by stimulating collateral artery development to bypass the partial coronary artery occlusion. Indeed, the EXCITE trial showed a significant improvement in coronary collateral flow index in response to 4 weeks of moderate- to high-intensity exercise [9]. In addition, Hambrecht et al. demonstrated that a 12-month training intervention resulted in a higher event-free survival rate and better exercise capacity compared to PCI [10]. To support the potential clinical benefits, a recent retrospective observational study in 18,383 patients with SAP showed that patients receiving exercise-based CR had 63% lower odds of all-cause mortality and 28% lower odds of acute myocardial infarction compared to PCI [11]. These observations highlight the potential of multidisciplinary CR as a primary treatment for SAP patients. Nevertheless, there have been no randomised controlled trials in the modern era that compared multidisciplinary CR to coronary revascularisation.

The first aim of this study is to compare the impact of a 12-month cardiac rehabilitation programme versus an invasive approach including coronary angiography and subsequent coronary revascularisation (i.e., usual care) in SAP patients with anginal complaints under OMT. We hypothesise that the 12-month CR programme will be at least equally effective as an invasive approach in reducing anginal complaints. The second aim is to evaluate the cost-effectiveness of the CR intervention compared to routine invasive care in SAP patients, as well as cardiovascular events, ischemic threshold during exercise, fitness, cardiovascular health, quality of life and psychosocial wellbeing.

## Methods/design

### Design

**Fig. 1:**
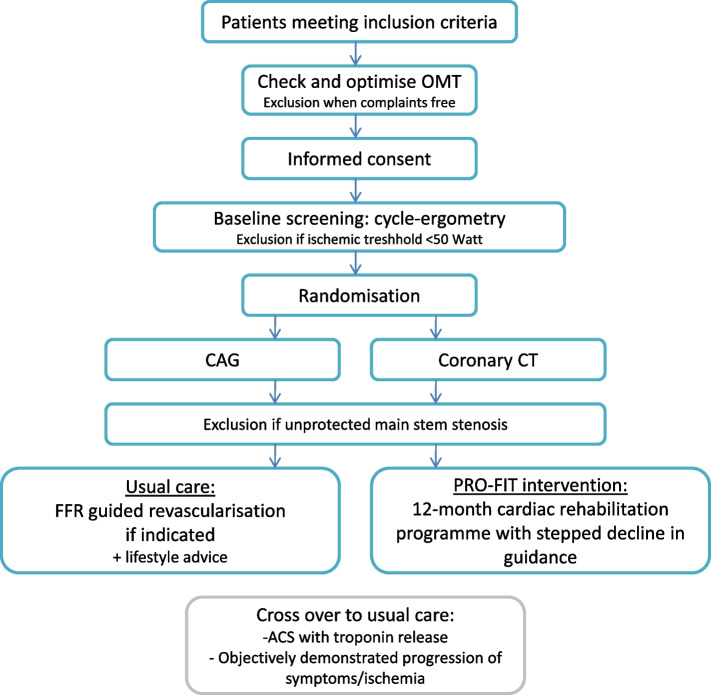
Flowchart of study inclusion process. ACS: Acute Coronary Syndrome; CAG: Coronary Angiography; Coronary CT: Coronary Computer Tomography; FFR: Fractional Flow Reserve; OMT: Optimal Medical Therapy

This study is designed as a multicentre randomised controlled non-inferiority trial. Patients will be included at the cardiology department of eleven participating Dutch hospitals (Additional file 1). Patients are randomly allocated to a 12-month cardiac rehabilitation programme (intervention group) or usual care (control group). A total of 216 patients will be included, each group will consist of 108 patients (Fig. 1). All subjects are requested to provide written informed consent before study entry. Data are collected at baseline and after three, six, nine, and twelve months. The study has been approved by an ethical committee (METC Oost-Nederland, registration number 2021–12,942) and has been registered in the Netherlands Trials Register (NL9537). SPIRIT reporting guidelines were used for writing the study protocol [12].

### Patient population and randomisation

The main inclusion criterion is a diagnosis of SAP with residual anginal complaints under OMT and at least moderate ischemia, assessed non-invasively within 12 months before inclusion (Table 1). In patients with SAP, both SPECT and cardiac MRI are common techniques to measure ischemia, with approximately 31–33% of the cardiac MRIs [13, 14] and 29–33% of de SPECT measurements being abnormal [13, 15]. Additionally, patients need to have access to a personal computer, laptop or tablet with internet connectivity at home and to a mobile phone with short message service (SMS) functionality. Exclusion criteria, mostly used to identify high-risk patients who might have a prognostic benefit from revascularisation, are shown in Table 1. Eligible patients will first receive an evaluation of their pharmacological treatment, which will be optimised according to the 2019 European Society of Cardiology Guidelines for the diagnosis and management of chronic coronary syndromes [16], including at least a beta-blocker, nitrate, and calcium channel blocker on a maximum tolerable dose unless contra-indicated (decision at discretion of the treating cardiologist). When asymptomatic after treatment optimisation, patients will be excluded from further study participation. Following inclusion, patients will receive an exercise test to exclude patients with an ischaemic threshold < 50 Watt. Finally, patients will be randomly assigned to either the intervention group (12-month CR intervention) or to the control group (usual care) using a 1:1 computerised randomisation system (Castor Electronic Data Capture 2021, Ciwit B.V., Amsterdam, NL) with block randomisation in random block sizes (range 4–6) for allocation concealment. After randomisation, patients will be screened for unprotected stenosis of the left main coronary artery (unless the coronary anatomy has been evaluated in the last 3 months) through computed tomography (intervention group) or a coronary angiogram (control group), see Fig. 1. If so, these patients will be excluded, as the latest guidelines present a class 1A indication for revascularisation, because of the prognostic benefit in terms of mortality [17].

**Table 1:** Inclusion and exclusion criteria PRO-FIT

**Inclusion criteria:**
1. Diagnosis of SAP with residual anginal symptoms after OMT
2. Established ischemia assessed by SPECT, PET, Stress echo, CMR, or cycle ergometry
3. Access to a personal computer, laptop or tablet with Internet connectivity at home
4. Possession of a mobile phone with short message service (SMS) functionality
**Exclusion criteria:**
1. PCI or CABG in the past year
2. Acute coronary syndrome in past 2 months
3. Angina symptoms at rest or rapidly progressive (i.e. unstable angina)
4. Ischemic threshold < 50 watts
5. Left ventricular ejection fraction < 35%
6. New-York Heart Association class III-IV heart failure symptoms
7. Advanced chronic kidney failure (i.e. estimated Glomerular Filtration rate < 30 ml/min)
8. Severe ventricular arrhythmia or exercise-induced arrythmia at baseline testing
9. A comorbidity precluding exercise training (e.g. orthopaedic, neurological or cognitive conditions) or other contra-indications for exercise training
10. Possible stenosis > 50% of the left main coronary artery on coronary CT of coronary angiography

### Control group

Patients in the control group will receive usual care, typically consisting of coronary angiography and, if appropriate, fractional flow reserve (FFR)-guided coronary revascularisation. Also, these patients will receive cardiovascular risk management and basic lifestyle advice according to local policy.

### Intervention group

**Fig. 2:**
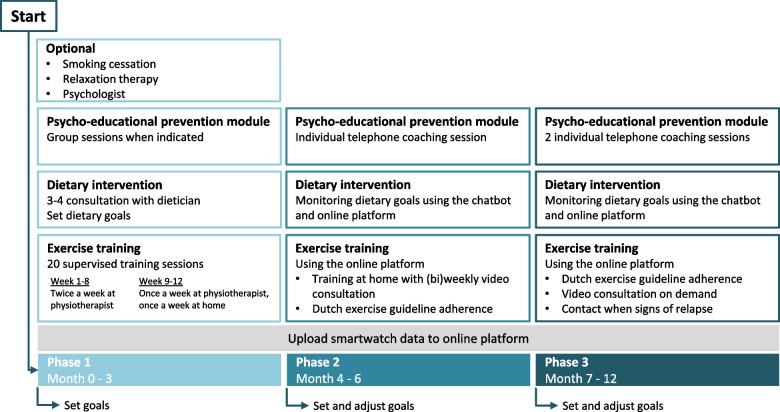
Cardiac rehabilitation programme overview

Patients in the intervention group will follow a 12 month CR programme aiming at angina relief and sustainable behavioural change for long-lasting improvement in cardiovascular health. The intervention is divided into three consecutive phases, with a stepped-care approach in decreasing the intensity of patients’ guidance. Phase 1 is the active supervision phase in which the main goals are behavioural change and improving exercise capacity and nutrition (month 1–3). Phase 2 consists of telerehabilitation with weekly to fortnightly video consulting and aims at integrating healthy behaviour in everyday life (month 4–6). Phase 3 focuses on relapse prevention and on-demand guidance (month 6–12). An overview of the CR programme is presented in Fig. 2.

To increase awareness and motivation and to help explore personal preferences with respect to lifestyle goals, patients are asked at baseline to complete a set of validated questionnaires on 6 lifestyle domains (body proportion, exercise and sedentary behaviour, smoking, alcohol, nutrition and stress), providing a response-based advice. During the intake procedure, a cardiac rehabilitation case manager (specialised nurse or sport physician) will assess individual rehabilitation needs on 5 domains: physical functioning, psychological functioning, social functioning, cardiovascular risk profile and risk behaviour [18]. Based on these needs and the patients’ individual preferences, individualised goals will be set by shared decision making, which represent the basis for the intervention.

#### Development of the PRO-FIT intervention

A consortium with experts from multiple domains was established to discuss and design this intervention. Specifically, we included the following experts; multiple cardiologists and physical therapists, a dietician, sports physician, medical psychologist, nutritional expert, medical physiologist, and an IT specialist. The study team was divided into several working groups, consisting of 7–11 individuals, to advise on the domain-specific content of the multidisciplinary CR intervention (physical exercise, nutrition, psychology and behavioural change, information technology and logistics). After 2–3 meetings, the evidence based aspects of the domain-specific intervention were integrated in a comprehensive CR programme. Details of this CR programme are described in the following paragraphs. Healthcare professionals responsible for delivering the CR programme were trained by the researchers and/or experts from the study team.

#### Online platform and activity tracking

A personalised, secured, patient-centred online platform with separate dashboards for patients and care professionals will be used for monitoring and coaching (FLOW platform, Mibida B.V., Eindhoven, The Netherlands). Patients are provided a smartwatch (Galaxy Watch Active2, Samsung, South Korea) at the start of the study to monitor heart rate during training sessions, including the time spent in the set heart-rate zone, and daily physical activity energy expenditure. Data will automatically be uploaded to the online platform by a secured cloud based solution.

The platform enables patients to register and adjust rehabilitation goals, review exercise training and activity data, monitor food intake with an integrated chatbot and conduct video consulting. Details of the platform were described earlier in a study protocol by Brouwers et al*.* [19]. Furthermore, it includes an integrated platform that promotes healthy behaviour through unified health gamification (GameBus, Eindhoven, The Netherlands) by involving the patients’ social environment and facilitating interaction with peers [20].

#### Exercise training

During phase 1 (month 1–3) all patients will follow an exercise training programme at a primary care physiotherapy practice. This exercise programme is designed to improve fitness, particularly by stimulating direct, exercise-induced effects on myocardial flow, endothelial function and arteriogenesis [21]. This will expectedly lead to improved coronary perfusion as demonstrated by previous studies in SAP patients [9, 22]. All physiotherapists are trained in cardiac rehabilitation and are part of *Chronisch ZorgNet* (a Dutch, national network of specialised therapists who offer supervised exercise therapy and lifestyle guidance). Patients will receive aerobic and resistance training, integrated in 20 face-to-face sessions. The aerobic training, consisting of continuous training, starts with a build-up phase in the first 2 weeks (40–50% heart rate reserve (HRR)), in which the patients get familiar with training. From week 3 forward the aerobic training intensity will be gradually increased to a target of 65–75%HRR. The resistance training will also start with a build-up phase in the first 2 weeks (30–40% 1 repetition maximum (1RM)) after which the training intensity will be gradually increased to a target of 50–80% 1RM. The training prescription is provided in Table 2. During phase 1, patients will receive education on how to incorporate exercise training and physical activity in their home environment. In achieving this goal, training data from their smartwatch will be used and visualised in the online platform for feedback purposes. From week 3 onwards, patients are stimulated to perform exercise training at home. The home-based exercise at this stage involves low-intensity exercise, with the moderate-to-high intensity exercise being restricted to the training sessions with the physiotherapist. From week 9 onwards, patients are instructed to also perform moderate-to-high intensity exercise training sessions at home using the target training intensity that is being adopted during the session with the physiotherapist. Frequency of this type of home-based training is once a week and intensity will be recorded using the smartwatch and online feedback. After 3 months, at the end of phase 1, the training goals of the patient will be evaluated and new or residual goals will be formed in consultation with the patient using the outcome measurements of this evaluation.

**Table 2:** Physiotherapy training prescription

*Training*	*Volume*		*Frequency*	*Intensity*	*Comments*
**Continuous aerobic training** week 1–2	- Warming-up	1 × 5–10 min	2/week	- 40–50% HRR - 55–60% W_peak_	- If no ischemic threshold: 60% max HR/47,5% HRR
	- Continuous load	2 × 10–15 min			
	- Cool-down	1 × 5–10 min			
**Continuous aerobic training** week 3–12	- Warming-up	1 × 5–10 min	2/week	- 65–75% HRR - 76–88% W_peak_	- If no ischemic threshold: 75% max HR/70% HRR - From week 4: increase with 10 min every 2 weeks
	- Continuous load	1 × 30–60 min			
	- Cool-down	1 × 5–10 min			
**Resistance training** week 1–2	- Warming up - 8–10 muscle groups (compound) - 1–3 sets - 10–15 repetitions (1–2 min break) - Cool-down		2/week	- 30–40% 1RM	- Repeat 1RM after 2 weeks
**Resistance training** week 3–12	- Warming up - 8–10 muscle groups (compound) - 1–3 sets - 10–15 repetitions (1–2 min break) - Cool-down		2/week	- 50–80% 1RM	- Week 3–10: Gradually increase intensity until 70–80% 1RM

*HRR* heart rate reserve, *W*_*peak*_ peak power output, *1RM* one-repetition maximum

- From week 9: 1 × exercise in home situation, 1 × supervised by physiotherapist

- Repeat 1RM every 4 weeks

The second phase (month 3–6) is aimed at the transfer of exercise training to the home environment. To this end, patients are advised to perform exercise training sessions in their home environment to reach their new or maintain previously set training goals with remote supervision. Patients are instructed to adhere to the Dutch physical activity guidelines 2017 [23] and perform two training sessions a week on a pre-set heart rate zone, which has been set in phase 1. In short, phase 2 exercise training will consists of:

- Two 40–60 min continuous aerobic training sessions a week on a pre-set heart rate zone with the modality adjusted to personal preference (e.g. cycling, walking/running)
- Two times a week muscle and bone strengthening activities as described in Table 2 under ‘resistance training’.
- Including the exercise above, all participants reach a minimum of 2.5 h/week moderate to vigorous intensity exercise, which is allowed to be spread across several days.

A video consultation of 10–20 min with a mentor from the core study team is scheduled every two weeks (or weekly, depending on patient preference) during this phase. During these video consultations, both the exercise data and, if appropriate, questionnaire results are evaluated and targets are adjusted if needed. These consultations are based on semi-structured interviews, in which the principles of motivational interviewing are applied [24–26]. Patients will be prepared for phase 3 and learn to adjust their own training and activity goals.

In phase 3 (month 6–12), we aim for sustained long-term effects to prevent progression of atherosclerosis. To achieve this, the intervention will contain a relapse prevention programme for six months after ending the second phase. Patients will be stimulated to continue performing exercise in their home environment, with the exercise goals set at the end of phase 2. In phase 3, coaching is only performed on an on-demand basis. The web application enables weekly evaluation of the exercise training and physical activity data and generates alerts in case of non-compliance (not uploading sensor data for 1 week), exceeded set heart-rate zones (> 20 min a week) and reduced physical activity. Reduced physical activity is defined as a decrease of 50% in total steps (average per week), a decrease of 50% in time spent in the moderate to vigorous activity zone (average per week), an exercise time that is 75% less than the Dutch Health Council guidelines or not attending or uploading two exercise sessions in a week. Coaching sessions can be initiated by caregivers to address the alerts or by patients if there are any questions.

#### Dietary intervention

Every patient will receive 3 consultations with a dietician within the first 3 months. Based on a dietary assessment at baseline, patients will get a personalised dietary advice following the Dutch Health Council guidelines for healthy nutrition and specifically focusing on cardiovascular health and reducing residual risk [27]. This assessment is done using the LifeStyleScore, which contains questions on 16 dietary domains derived from the Dutch Health Council guidelines. The dietary assessment will be repeated every 3 months to set new or adjust existing nutritional goals. To stimulate adherence to a healthy diet, a chatbot will be used. This chatbot will monitor the progress on personal goals and increases patients´ awareness by asking about their intake and showing the results in the online platform. A full day recall will be assessed at least every five days, up to daily, depending on a patient's personal preference.

#### Information module

Every patient will receive access to an information module. This module consists of 10 short videos, recorded by professionals, containing information about several relevant topics such as clinical trial information, coronary artery disease, medication and a healthy lifestyle (e.g. exercise, nutrition, smoking cessation, stress, work, maintaining a healthy lifestyle).

#### Psycho-educational prevention module (PEP)

The lifestyle PEP module is a structured behavioural change programme that uses psychoeducation and evidence-based techniques in guiding patients towards their goals. These techniques include; cognitive behavioural therapy, motivational interviewing, solution focused therapy, acceptance and commitment therapy, positive psychology and mindfulness. The programme focuses on the patients’ preferences and on aspects in their lifestyle that can be altered to reduce the risk of recurrence and/or worsening of cardiovascular disease. In general, these themes are exercise, smoking, weight loss, alcohol, nutrition, and stress (BRAVO themes). This is in accordance with the Dutch CR guideline [28]. The PEP module consists of three individual telephone coaching sessions with a lifestyle coach (month 5, 7 and 9) and, if indicated, also of PEP group sessions in month 1–4.

#### Psychological counselling and relaxation therapy

At baseline, a psychological screening is performed through a combination of questionnaires assessing symptoms of depression, anxiety and stress according to the Dutch Society for Cardiology CR-guideline [18]. If indicated, patients are referred to relaxation therapy or to a registered psychologist for individual treatment.

#### Smoking cessation

If patients smoke they are referred to a smoking cessation programme based on group training, telephonic coaching or personal coaching, based on the patient’s preference.

### Primary outcome measure

#### Angina symptoms

The primary outcome measure is the change in angina symptoms from baseline to 12 months, which will be compared between the intervention and control group to answer the main research question. The quantity of angina symptoms will be evaluated by the shortened Seattle Angina Questionnaire (SAQ-7) [29]. The SAQ-7 has good validity and reproducibility in patients with angina pectoris and correlates with prognosis [29]. Furthermore, through its limited length it can easily be used in routine clinical practice.

### Secondary outcome measures

#### Clinical outcomes

Secondary clinical outcome measures include ischemic threshold, major adverse cardiac events, health-related quality of life, physical fitness, cardiovascular health, psychosocial wellbeing and physical activity pattern. See Table 3  for the assessment schedule.

1. Ischemic threshold is evaluated at baseline and 1 year and is defined as the exercise intensity (Watts) at which ECG abnormalities occur during symptom-limited exercise testing. If no ischemia is observed at follow-up, the maximal achieved workload in watts will be used. Ischemia is defined as at least 1.5 mm ST-segment depression in ≥ 2 leads or ≥ 2 mm ST-segment depression in a single lead at < 7 metabolic equivalents. This measure is frequently used to examine the impact of (non)pharmacological strategies in SAP patients and is strongly linked to clinical outcomes [30]. The ischemic threshold of every exercise test will be assessed by two cardiologists from the study team who are blinded for randomisation. All patients perform a supervised symptom-limited exercise test on a cycle ergometer, using an individualised ramp protocol with duration of 8 to 12 min. Although being individualised, the protocol will be kept similar within individuals. The test is ended when a patient is not able to maintain the required pedalling frequency of 70 per minute. A 12-lead electrocardiogram is registered continuously.
2. Major adverse cardiovascular events are evaluated using the International Classification of Disease-10. We will record occurrence of death of cardiac cause, non-fatal myocardial infarctions, (repeated) myocardial revascularisations (specified to urgent and non-urgent) and unplanned hospitalisation owing to worsening angina. Revascularisation is considered to be urgent when a patient is admitted to the hospital with persistent or increasing chest pain (with or without ST-segment or T-wave changes or elevated biomarker levels) and the revascularisation procedure was performed during the same hospitalisation. These data are extracted from the electronic patient record at 1-year follow-up. This will be double checked with the patient during the last (12-month) study visit.
3. Health related quality of life is evaluated at baseline, 3, 6, 9 and 12 months, using the EQ-5D-5L questionnaire in a validated Dutch version [31].
4. All participants perform a supervised symptom-limited exercise test at baseline and 12 months. To examine physical fitness, we also record peak workload, expressed in watts which represents a surrogate for physical fitness level.
5. To examine cardiovascular health, general cardiovascular risk factors are assessed at baseline, 3 and 12 months. This includes blood pressure, lipid profile, haemoglobin A1c, fasting glucose and body-surface measurement (weight, length and body-mass index).
  - Blood pressure measurements is performed 3 times, with a 5 min interval, at the arm with the highest value, by a heart-function technician or a member of the research team. The mean systolic and diastolic pressure is calculated.
  - Lipid profile is evaluated by blood tests of total cholesterol, low-density lipoprotein, high-density lipoprotein, triglycerides and lipoprotein-ratio. Blood tests are performed at the patient’s own hospital and extracted from the electronic patient file.
  - Haemoglobin A1c and fasting glucose are measured by blood tests. HbA1c levels are strongly associated with major adverse cardiovascular events and severity of coronary artery disease in patients with and without diabetes [32, 33].
  - Body-surface measurement is performed through measurement of weight (kilogram) and length (meters) and expressed in kg/m2.
6. To evaluate psychosocial wellbeing, both anxiety and depression are assessed at baseline, 3 months and 1 year using the HADS-questionnaire consisting of a depression subscale (HADS-D) and an anxiety subscale (HADS-A). This questionnaire is advised by latest guidelines which also provide cut-off values for the screening on anxiety and depression [18].
7. To gain insight into lifestyle changes of both groups, sedentary behaviour and physical activity are assessed at baseline and 12 months, using the validated Sedentary behavior questionnaire (SBQ) [34] and Short questionnaire to assess health-enhancing physical activity (SQUASH) [35]

**Table 3:**
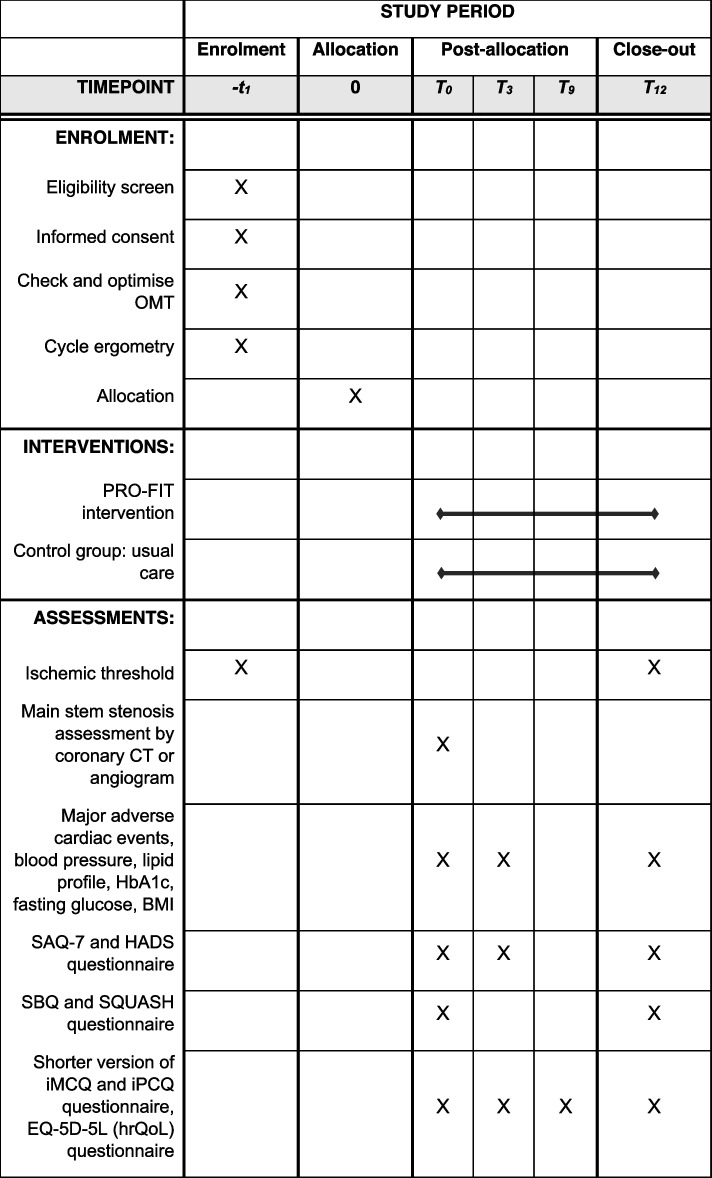
Schedule of enrolment, interventions and assessments

*BMI* Body mass index, *Coronary CT* Coronary computer tomography, *EQ-5D-5L* EuroQol 5-level EQ-5D, *HADS* Hospital Anxiety and Depression Scale, *HbA1c* Haemoglobin A1c, *hrQoL* Health related quality of life, *iMCQ* imta Medical Cost Questionnaire, *IPCQ* imta Productivity Cost Questionnaire, *OMT* Optimal medical therapy, *SAQ-7* Seattle angina questionnaire-7, *SBQ* Sedentary behaviour questionnaire, *SQUASH* Short questionnaire to assess health-enhancing physical activity

#### Cost analysis

For the second aim of this study, a cost analysis, cost-utility analysis and budget impact analysis will be performed at baseline, 3, 6, 9 and 12 months, comparing the intervention- and control group.

(8) To perform the cost analysis, we will first examine the direct and indirect medical and (for the societal scenario) non-medical costs. The direct and indirect medical costs include all costs of inpatient and outpatient hospital visits, diagnostic and therapeutic procedures and consultations. The data will be collected digitally from hospital information systems and with clinical report forms. The costs of out-of-hospital care by general practitioners as well as the direct non-medical out-of-pocket expenses (over-the-counter medication) will be based on volume data gathered with repeat patient questionnaires at baseline, three, six, nine, and twelve months post randomisation. This questionnaire will be adapted to the patient group and is a shorter version of the iMCQ [36]. Unit costing will preferably be based on the existing national guideline for costing in health care research [36]. Otherwise, the unit costs especially those for hospital admissions will be assessed using hospital based absorption costing. Unit costs derived from different calendar years will be indexed to 2019 prices. Indirect cost, e.g. productivity cost, will be based on the friction cost approach, according to guideline. For the indirect cost, a shorter version of the iPCQ questionnaire will be used [36].
(9) To perform the cost-utility analysis, insight into health-related quality of life is needed, which is recorded by a generic quality of life questionnaire. For a health related quality of life generic measure, we use the EQ-5D-5L questionnaire in a validated Dutch version [31].The questionnaire is held at baseline, 3, 6, 9 and 12 months of follow-up to generate health status scoring profiles over time, which will be transposed into health utilities using population based tariffs of time trade-off ratings of health states.
(10) The budget impact analysis makes use of the cost information of the cost effectiveness analysis. The primary aim of this budget impact analysis is to assess the financial consequences and affordability of nationwide implementation of the comprehensive CR intervention in patients with SAP from the budget holder’s perspective (Budgettair Kader Zorg). A medium term evaluation (up to three years) will be performed based on extrapolation of the empirical findings (Cost analysis) possibly combined with secondary evidence. We will use the framework as presented in the Dutch guideline for economic evaluations [36] and we globally adhere to the ISPOR (International Society for Pharmacoeconomics and Outcomes Research) guidelines [37]. The short- and mid-term affordability of the multi-facet strategy will be assessed from different scenarios: governmental, health care (Budgettair Kader Zorg) and insurer perspectives.

### Sample size calculation

The sample size was calculated based on data from the ISCHEMIA trial, in which similar in- and exclusion criteria for SAP patients were applied as in our study [38]. The primary analysis will be based on the comparison between the two treatment arms at 12-month follow-up of the mean SAQ Summary score. The sample size assessment is based on comparing groups based on the 95% two-sided confidence interval for independent means, adjusted for baseline values (ANCOVA). The adjustment for baseline will provide an efficiency gain of 16% compared to an independent t-test, assuming a correlation of 0.4 (data from ISCHEMIA trial). The standard deviation for the SAQ summary score at 12-months is assumed to be 19 points (also data from ISCHEMIA trial). With a correction of 10% drop-out, 7% sample size gain will remain. Aiming for a power of 80% and under these assumptions, assuming equal means for both groups and a non-inferiority margin of 7 points, the required sample size is 195 patients. Accounting for 10% dropout, a total of 216 patients needs to be included.

### Statistical analyses

To test non-inferiority in reducing anginal complaints, the primary analysis will be based on the comparison of mean SAQ-score at one year follow-up between both study arms, with an intention-to-treat ANCOVA analyses. The ANCOVA analysis will be adjusted for baseline SAQ-score values and sex. In case of cross-over, meaning that a SAP patient in the intervention group requires revascularisation, the last available SAQ Summary score before the switch will be used. This is expected to be a conservative measure for demonstrating non-inferiority. Non-inferiority is proven if the upper limit of the 95% two-sided confidence interval of the mean difference between the two groups does not exceed the non-inferiority margin of 7.

Secondary endpoints will also be analysed using an intention-to-treat analysis. Differences between the two groups will assessed using ANCOVA for continuous variables and logistic regression for dichotomous variables.

Categorical baseline variables will be presented as frequencies and percentages, continuous variables as means and standard deviations, or means and interquartile ranges for variables with skewed distributions. Percentages will be calculated on the number of non-missing observations. In all cases the number of missing values will be specified.

#### Cost analyses

For the cost analysis, the primary analysis will be based on the comparison between the two treatment arms. Cost is usually a parameter with a skewed distribution. If this occurs, as well as possible heteroscedasticity, cost analysis will be performed using a generalised linear model with a log ink function relating the conditional mean to independent variables and using a gamma distribution specifying the relationship between the variance and the mean.

For the utility analysis, health utility scores over time will be used. Quality adjusted life years will be calculated by taking the product sum of the health utility scores and the periods in-between successive measurements during the 12 months of follow-up according to the trapezium method. Utilities will be analysed using tobit regression.

The cost-utility analysis integrates costs and utilities to an Incremental Cost-Effectiveness Ratio with bootstrapped confidence intervals surrounding the point estimate of this ratio.

Analysis will be carried out in the statistical software package R.

## Discussion

The PRO-FIT study is the first study directly comparing the impact of a conventional invasive approach versus a multidisciplinary cardiac rehabilitation programme as a primary treatment in SAP patients on anginal complaints. The CR intervention is multidisciplinary and includes exercise training to improve coronary perfusion and behavioural change strategies in combination with modern technology to optimise long-term adherence to a healthier lifestyle and thereby reduce cardiovascular morbidity. The objective of this study is to investigate whether a comprehensive CR-intervention is at least equally effective in reducing anginal symptoms as a conventional, invasive approach.

In 2004 Hambrecht et al*.* highlighted the potential benefits of exercise training in SAP [10]. In this randomised controlled trial, 100 patients with an angiographically objectified stenosis were randomly assigned to PCI or a 12-month exercise intervention. The training intervention resulted in a higher event-free survival rate and better exercise capacity after 12 months compared to PCI. The results were achieved using exercise training only, and therefore may even underestimate the true potential of lifestyle changes for this patient group. Past years, studies have demonstrated the benefit of a multimodality programme over exercise training only as multimodality programs impact several levels of a healthy lifestyle [39, 40]. By offering SAP patients a multidisciplinary CR programme, addressing other risk behaviour as well, the clinical benefits may be superior to exercise training only. On the other hand, the development of drug eluting stents and the advancement of antiplatelet therapy improved the clinical outcomes of invasively treated patients in the period following Hambrecht’s trial [41–43]. Given these advancements, results of our study and Hambrecht’s trial might not be corresponding.

Besides the potential benefits of CR as a first line treatment for SAP on clinical outcomes, CR may also be more cost-effective than invasive treatment. A first indication for this hypothesis is provided by Hambrecht’s CR intervention, which demonstrated CR to be more cost-effective than PCI [10]. As health care costs are mounting, primarily by a growing population and an unhealthy lifestyle, low-cost alternatives to invasive procedures are important to reduce the burden of stable cardiovascular disease, both for patients as for society. If CR demonstrates to be effective, it is important to realise that CR is also scalable and affordable. For this purpose, we aim to conduct the CR intervention through first line physiotherapists using an already existing network with nationwide coverage. If proven successful, this approach allows for successful nationwide implementation and will lower the pressure on hospital resources and staff. Furthermore, our CR programme has an early transition from centre-based CR to telerehabilitation to improve cost-effectiveness, as previous studies showed lower social costs in telerehabilitation, particularly due to lower absenteeism [44, 45].

The Ischemia trial already showed that revascularisation does not reduce the risk of ischemic cardiovascular events or death from any cause compared to OMT alone, but does reduce anginal complaints. Our study is designed to investigate whether OMT supplemented with a comprehensive CR-intervention reduces anginal complaints just as effectively as revascularisation. There is a strong, (patho)physiological rationale underlying this concept. By choosing PCI as the primary target, treatment is limited to addressing a local obstruction only. However, SAP patients often demonstrate poor vascular health at systemic level, and are at increased risk of developing cardiovascular events later in life [9]. The local focus of PCI does not improve the overall endothelial function nor prevent future obstructive plaques causing subsequent procedures. Therefore, a lifestyle intervention, aiming to improve both local perfusion and function of the entire vascular system, might be a better alternative to a local, invasive strategy in these patients.

Replacing invasive interventions with lifestyle interventions is not common in contemporary medicine, although some examples exist. Patients with peripheral artery disease for example, are currently referred for first line supervised exercise training as a (cost-)effective treatment, whereas invasive vascular interventions to target the local stenosis used to be standard treatment upon presentation of complaints. Exercise training demonstrated superior effects on intervention-free survival, walking distance and quality of life compared to invasive vascular interventions [46, 47]. Following this evidence, international guidelines recommend exercise training as a treatment of first choice in patients with intermittent claudication [48]**.** In analogy to stable angina, these studies highlight that addressing the systemic vascular problem, rather than focusing on the local lesion, may improve clinical outcomes. In relation to stable angina, the recent retrospective observational study by Buckley et al*.* supports this concept and emphasises the potential of CR as a treatment for SAP patients, showing a significant reduction in all-cause mortality and myocardial infarction compared to PCI [13]. This clinical example around peripheral artery disease also highlights the importance of studies that directly compare invasive *versus* non-invasive strategies on clinically relevant outcomes.

In conclusion, we hypothesise that multidisciplinary CR is at least equally effective in reducing anginal complaints as an invasive approach in patients with stable angina pectoris. This comparison is highly relevant, since CR may be related to lower costs (and complication rates) compared to PCI, whilst CR is aimed at improving systemic health status and lowers risks for other cardiovascular diseases. If proven successful, this study will have a significant impact on the daily care of these patients as coronary revascularisations can largely be replaced by multidisciplinary CR; a less invasive, less costly and better sustainable treatment.

### Trial status

The inclusion of patients has started in February 2022. As of April 2023, we have enrolled 38 patients in the study. Recruitment will continue until the complete sample size is achieved.

## Supplementary Information

**Additional file 1.** Participating hospitals.

## Data Availability

Not applicable.

## Abbreviations

ANCOVA: Analysis of covariance
CAG: Coronary angiogram
CMR: Cardiac magnetic resonance imaging
CR: Cardiac rehabilitation
EQ-5D-5L: EuroQol 5-level EQ-5D
FFR: Fractional flow reserve
HADS: Hospital Anxiety and Depression Scale
HbA1c: Haemoglobin A1c
HRR: Heart rate reserve
IMCQ: Imta Medical Cost Questionnaire
IPCQ: Imta Productivity Cost Questionnaire
OMT: Optimal medical therapy
PCI: Percutaneous coronary intervention
PEP: Psycho-educational prevention module
PET: Positron emission tomography
SAP: Stable angina pectoris
SAQ: Seattle angina questionnaire
SBQ: Sedentary behaviour questionnaire
SMS: Short message service
SPECT: Single photon emission computed tomography
SQUASH: Short questionnaire to assess health-enhancing physical activity
Wpeak: Peak power output
1RM: One-repetition maximum
