# Supplementary material for: Impact of a comprehensive cardiac rehabilitation programme versus coronary revascularisation in patients with stable angina pectoris: study protocol for the PRO-FIT randomised controlled trial
Source: BMC Cardiovasc Disord. 2023 May 5;23:238. doi: 10.1186/s12872-023-03266-z (PMC10163688; doi:10.1186/s12872-023-03266-z)
Supplement: Supplementary file 1 — Additional file 1. Participating hospitals. [file 12872_2023_3266_MOESM1_ESM.docx]

**Participating hospitals**

Radboud University Medical Centre, Nijmegen; Maxima Medical Centre Veldhoven/Eindhoven; Catharina Hospital, Eindhoven; Maastricht University Medical Centre+, Maastricht; Elkerliek Medical Centre, Helmond; Medisch Spectrum Twente, Enschede; Franciscus Gasthuis & Vlietland, Rotterdam; Diakonessenhuis, Utrecht; Amsterdam University Medical Centre (location Academic Medical Centre), Amsterdam; Ikazia Hospital, Rotterdam; Maasstad Hospital, Rotterdam.
